# The HeDiCom framework: Higher Education teachers’ digital competencies for the future

**DOI:** 10.1007/s11423-023-10193-5

**Published:** 2023-03-22

**Authors:** Jo Tondeur, Sarah Howard, Manon Van Zanten, Pierre Gorissen, Irma Van der Neut, Dana Uerz, Marijke Kral

**Affiliations:** 1 Interfaculty Department of Teacher Education, Vrije Universiteit Brussel, Pleinlaan 9, Brussels, Belgium; 2 School of Education, University of Wollongong, Wollongong, Australia; 3 HAN University of Applied Sciences, Arnhem, The Netherlands; 4 IVA Onderwijs, Tilburg, The Netherlands

**Keywords:** Curriculum analysis, Digital competencies, Higher Education, Technology curriculum, ICT frameworks

## Abstract

There is little consensus about the nature of teachers’ digital competencies in Higher Education. Moreover, existing digital competence frameworks have largely been developed for teachers in secondary education. In response to this, the current study focuses on developing and validating a framework of digital competencies for teachers in Higher Education. First, a review was conducted to determine the state of digital competence research regarding dimensions and definition of digital competence. In a next step, similarities and differences between existing digital competence frameworks were identified. Based on the outcomes of the review and the framework comparison, a framework was developed in an iterative process through expert meetings with policy makers, experts in the field of educational technology, and validated with practitioners. The new framework includes four dimensions of teachers’ digital competencies: (1) Teaching practice, (2) Empowering students for a digital society, (3) Teachers’ digital literacy, and (4) Teachers’ professional development. The resulting Higher Education Digital Competence (HeDiCom) framework will provide guidance and clearer expectations of teachers’ digital competency. Ultimately, improving teachers’ digital competencies will contribute to improving the quality of digital competencies of the students.

## Introduction

With the rapid increase of technology available to support learning in recent decades, developing the digital competence of university graduates to prepare them for the future workforce has become a priority. However, for these digital competencies to be developed in Higher Education (HE) students, their learning environments and tasks must be designed to provide opportunities to build these competencies. Clearly, HE teachers must be able to organize learning environments in which students themselves develop digital competencies. This is particularly important in relation to online and blended learning. The Covid-19 pandemic has forced teachers in Higher Education towards a rapid and massive shift to online teaching across the world (Schleicher, 2020). Teachers, most of whom had never fully taught online, were asked to redesign their teaching practice to support their students in an online environment (see Scherer et al., 2020). The question remains to what extent these teachers are prepared to teach online or in a blended setting.

Teaching with digital technologies requires digital competencies but also different pedagogical approaches than for instance teaching face-to-face (Gurley, 2018). As a consequence, the focus on digital competence continues to grow in popularity in Higher Education (Zhao et al., 2021). Clearly, teachers are expected to adequately use digital technologies to strengthen their teaching practice and enhance their educational practice. The problem is that it is not always clear which digital competencies HE teachers should possess to adequately integrate ICT into their educational practice, and they lack guidance on developing their digital competence (Basilotta-Gómez-Pablos et al., 2022; Bennett, 2014; Tondeur et al., 2018).

There are a number of digital competency frameworks created by government agencies and nonprofits (e.g., ISTE, DigcompEdu), but these have mainly focused on teachers in school contexts rather than Higher Education. To complicate this issue, there is little consensus about what features should be included in a HE competency framework (Tondeur et al., 2018). The current study intends to provide practitioners, the research community, as well as policy makers with a digital competence framework that can be used in Higher Education. This would provide important guidelines for teachers in Higher Education on implementing and integrating ICT in their teaching practices, promoting innovation, and sustaining professional growth. It also signals support that may be needed to develop digital competencies. In a first step we reviewed the evidence based on the digital competencies teachers in Higher Education should possess. Next, we identified similarities and differences between existing frameworks, to act as a lever for the development of necessary digital competencies to form an initial framework. In a final step, we validated the initial framework in order to refine the framework on the basis of experts’ opinions and to explore the recognizability and usability of the framework for institutions of Higher Education. The result of this work has been a new framework for digital competence in Higher Education, which provides a straightforward tool for direct implementation in HE teaching.

## Background

The notion of digital competence is not new in educational research and training. However, competencies have changed and evolved over time relative to political, social, and educational contexts (Ilomäki et al., 2016). Digital competence has been understood in relation to digital literacy, digital capabilities, digital knowledge, etc. In the following section we first address digital competence and how it has been defined, then consider existing frameworks and their relation to Higher Education.

### Digital competence

Digital competence is a critical element in a teacher’s successful integration of digital technologies in learning (Tondeur et al., 2018). It is of particular importance when considering how teachers move to designing online, blended or hybrid learning spaces. Throughout the years, different terms have been used to capture ‘digital competence’, namely it has overlapped with ‘digital literacy’ in terms of higher order capabilities, such as problem solving with digital technologies. However, competencies have typically combined digital skills with digital literacies. As such, in 2002 the OECD began work to identify key competencies in training and education and a way to understand these competencies across contexts. They defined competency as “the ability to meet demands or carry out a task successfully, and consists of both cognitive and non-cognitive dimensions” (OECD, 2002, p. 4).

In the case of digital competence, cognitive aspects refer to digital literacies, while non-cognitive dimensions are more closely related to digital knowledge, capacities and efficacy. As this definition evolves, the enduring aspect of competencies is the successful use of technologies to meet demands—whether these be social, work or learning. In the current study, we follow the definition of ‘digital competence’ used by the General Secretariat of the Council of the European Union. This definition provides a comprehensive view of the role of digital technologies and digital competence in education:

Digital competence involves the confident, critical and responsible use of, and engagement with, digital technologies for learning, at work, and for participation in society. It includes information and data literacy, communication and collaboration, media literacy, digital content creation (including programming), safety (including digital well-being and competences related to cybersecurity), intellectual property related questions, problem solving and critical thinking. (European Union, 2018).

### Digital competence and Higher Education

Developing digital competencies of students to prepare them for the future workforce has become a priority in many universities around the world (Tondeur et al., 2018; Zhao et al., 2021). Higher Education institutions are “rich in technology resources and technology-based activities” (Selwyn, 2010, p. 2), but the risk of digital divisions among students in regard to what they use and how they use it is high. Moreover, it is expected that students will soon enter the workforce and there is a need for them to perform as knowledge workers, which requires a high level of digital competence (Ilomäki et al., 2016). For students to achieve a high level of digital competencies in their field, to become successful knowledge workers, HE teachers must also possess the digital competence to guide their development. Zhao et al. (2021) have shown that HE teachers and students have basic digital competence; however, without clear standards of digital competence developing these competencies remain elusive. According to Zhao et al. (2021), the most frequently identified competencies were the use of digital technologies, knowledge of digital technologies, the Internet and technological related capacities, digital experiences and attitudes.

The competencies mentioned above have been identified in research, but existing digital competency frameworks, such as DigcompEdu have been created for use at the compulsory school level (see Redecker & Punie, 2017). In some cases, research has drawn on school-focused competency frameworks (Zhao et al., 2021), but the skills, attitudes and experiences of school learners and teachers, when compared to Higher Education, are quite different. Frameworks specifically designed for Higher Education are needed to address the complexity of digital technologies in the context and trajectory to the workplace. In this respect, Lin and Johnson (2021) have called for more research that is directly applicable to the specific teaching and learning context. A key aim of the current research has been to create a framework that can be used by HE teachers, to reflect on their own practice. Existing frameworks, while comprehensive and providing rich definitions of competencies, have also been at a level of detail that can be difficult for practical implementation to support the ongoing digital shift in education (see Howard et al., 2022), especially since the outbreak of the COVID-19 pandemic. This Great Online Transition (Scherer et al., 2021; Tondeur et al., 2021) has transformed most HE from traditional to online and blended teaching and learning (see also Zarei & Mohammadi, 2022). Moreover, HE institutions that cultivate elites for our society, should be committing to innovate and to effectively integrate educational technologies (Cordie & Lin, 2018). As a result, this HE context requires education institutions to re-consider the necessary digital competencies. This brings us to the purpose of the study.

### Context and purpose of the study

As stated in the literature, a debate exists concerning the nature of teachers’ digital competencies and how they can be best developed in Higher Education (cf. Falloon, 2020). The majority of digital competence frameworks were developed specifically for compulsory education. Only recently has the field turned its attention to competencies in Higher Education. But according to Zhao et al. (2021) “it is still not easy to get a full picture of digital competence of teachers and students in the context of Higher Education” (p. 6). The purpose of this study was to create a framework for digital competencies in Higher Education. Such a competence framework can serve as a common reference (Vuorikari et al., 2016) by depicting articulated digital competencies and hence support the development of expertise (McGee et al., 2017). In that way, a comprehensive framework is needed that can improve the transparency and simplify what is expected of teachers. Ultimately, improving the digital competencies of teachers will also enhance the quality of the educational activities and the digital competencies of the students.

## Research design

The aim of this study is the development and validation of a framework of digital competencies for teachers in Higher Education. This was done in three stages. The first stage comprised a literature review which was conducted in order to provide an objective and comprehensive view of the existing literature and frameworks. The main goal of this review was to provide an overview of competencies relevant to teaching and learning with ICT in Higher Education. The second stage included the analysis of existing digital competence frameworks to establish main- and sub-dimensions of digital competencies, determining similarities and differences between the frameworks. The result of these two stages was an initial draft framework for digital competencies in Higher Education. In the final stage, the initial framework was reviewed by experts and refined. The revised framework was then validated in a series of focus groups with practitioners. Below we present the procedure in more detail.

### Review of the literature

Between December 2020 and February 2021, a review of literature on digital competencies was conducted to locate, critically evaluate, and synthesize studies about the teachers’ digital competencies. This study drew on a systematic review approach, which is defined as an interpretation of a selection of documents on a specific topic that optimally involves summarization, analysis, evaluation, and synthesis of the documents (Petticrew & Roberts, 2006). Four inclusion criteria were employed: (1) the language of the article is English, (2) the article was published between December 2010 and December 2020, (3) only articles and reviews are accepted, no other type of publication, (4) the article is peer-reviewed in the database Web of Science. The search terms used were: (ICT) AND (Higher Education) AND (Competence*) AND (Lecturer OR Educator OR Teacher OR Faculty OR Professor). Initially, 122 empirical articles were identified. Abstracts were examined to identify the educational level and if the topic was relevant for the review, which identified 36 relevant articles. The excluded articles had, for example, a focus on teachers’ perception on their digital competence or on their level of digital competence, without discussing what competencies were included in the digital competence. Full-text readings of these articles further identified 21 articles that were specifically addressing digital competencies of teachers in Higher Education. These articles were then synthesized.

### Comparison of existing frameworks

The following frameworks were compared: Competence framework for Teaching and Learning with ICT (van Loon et al., 2018), DigCompEdu (Redecker & Punie, 2017), Digital Teaching Professional Framework (Education & Training Foundation, 2019), ISTE Standards for Lecturers (ISTE, 2017), JISC Teacher Profile (JISC, 2019) and UNESCO ICT competency framework for teachers (UNESCO, 2018). The main and sub-dimensions of the frameworks were identified, existing similarities were merged where necessary, and the remaining digital competencies were mapped. The result was an overview of identified dimensions. This resulted in an initial draft of the HeDiCom framework, which included four main themes: Teachers’ digital literacy, Teachers’ Professional Identity; Teaching and Learning with Technology; Empowering students. The overview further provided a list of possible main-and sub-dimensions, related to these themes. The initial framework was further refined through expert discussions (see section “Expert meetings and validation of the framework”).

### Expert meetings and validation of the framework

Experts participating in the study were identified as Dutch-speaking senior academics working in the area of digital technologies and teacher education, in Higher Education. Expert meetings comprised 11 experts across three groups, which met online twice over two months. In the first meeting, each group discussed design criteria for the framework, the initial draft of the framework with its themes, and dilemmas at hand. The main design criteria were: the framework should be specifically aimed at educational digital innovation in Dutch Higher Education Institutions; it should include competencies on empowering students for a digital society, with special attention to students’ future profession; and the framework should be recognisable and useful for educational professionals in Higher Education and support them in improving their educational practice. The framework was then refined and again reviewed in an iterative manner in each subsequent session. In this process, main- and sub-dimensions were further defined. This resulted in a complete first version of the new framework for digital competencies. The resulting first version of the framework was then validated through online sessions with 34 educational professionals and teachers.

## Results

**Fig. 1:**
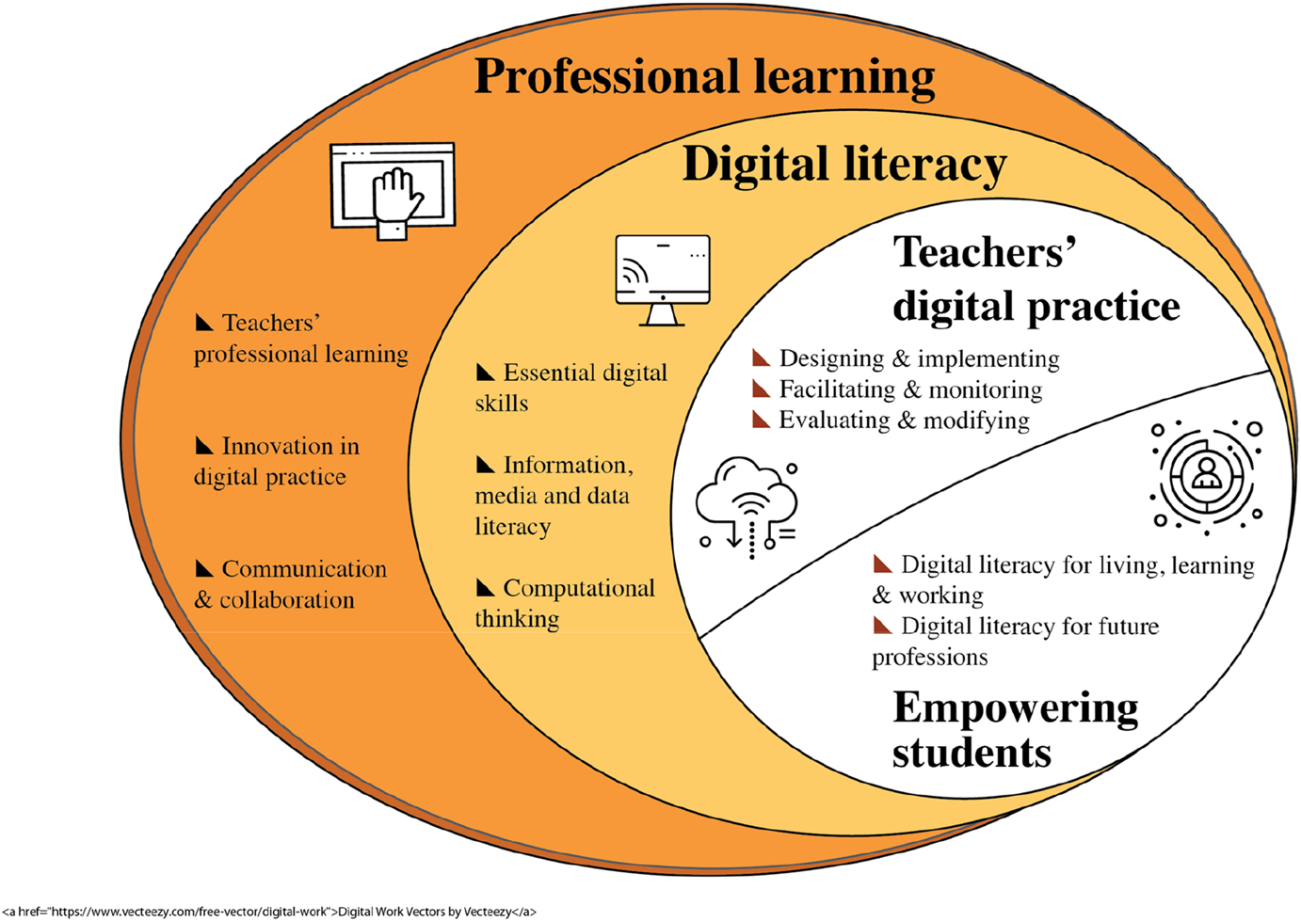
The HeDiCom framework

The final version of the Higher Education Digital Competence (HeDiCom) framework includes four dimensions, with two or three subdimensions (see Fig. 1). In the next section, we will discuss the four main dimensions, its subdimensions and the associated competencies.

### Teachers’ digital practice

The first dimension of the framework relates to digital competencies that are required as part of teachers’ digital practice. The (re)design of digital learning requires specific competencies of teachers (Basilotta-Gómez-Pablos et al., 2022; Bennett, 2014). The findings of the literature review emphasize that, in the context of stimulating educational change through the use of ICT, it is important for teachers to be aware of how and why they want to integrate digital technologies in a specific context, while ensuring alignment between learning objectives, learning activities, learning resources and assessment (see Heitink et al., 2016). As a consequence, integrating ICT into education requires teachers in HE to rethink their educational designs, implement new or refined designs, evaluate the results and then potentially re-design, beginning the process again. Therefore, the three sub-dimensions are: (1) Designing and implementing, (2) Facilitating and monitoring, and (3) Evaluating and modifying.

#### Designing and implementing

The findings of the literature review point to the importance of the sub-dimension “Designing and implementing” (e.g., Ardiç & Çiftçi, 2019; Cabero-Almenara et al., 2020; Romero Alonso et al., 2019). Some studies also highlight that ICT can act as a catalyst for innovation in practice, for example to create more flexible, personalized and self-regulated learning (Schneckenberg, 2010). As part of the Great Online Transition (GOT) (Scherer et al., 2020), teachers in HE have needed to transform their teaching practice into remote, online and blended learning formats (Scherer et al., 2021; Tondeur et al., 2021). To become a competent online teacher, there is a need for professional development and sufficient time to design and practice online and blended learning environments (see also Kebritchi et al., 2017). To do this, it is important that HE teachers create new digital educational resources, change and arrange existing sources (e.g., Cabero-Almenara et al., 2020). Some studies emphasize the potential of including students in the design process and implementation of these new designs (ISTE, 2017; Redecker & Punie, 2017) while also taking into account student well-being and social inclusion. This is an aspect that receives very little attention in the literature review on teachers’ digital competencies, but it is mentioned in several frameworks (cf. DigCompEdu and JISC).

#### Facilitating and monitoring

When using ICT to facilitate and monitor student learning, the second sub-dimension, HE teachers should make conscious use of the possibilities offered by ICT to improve or support students’ learning. Teaching should be aligned with the needs of students, for instance to ensure more flexible and personalized learning and greater student self-regulation (Cabero-Almenara et al., 2020) and to facilitate collaborative learning (Ricardo-Barreto et al., 2020). Teachers also need to be able to use digital technologies for student assessment. Different formative and summative assessment strategies using ICT can increase the effectiveness of assessment, for instance to give online feedback or peer review (Segovia Cifuentes & Díaz Gómez, 2016). The literature review reveals that the data generated by various systems can be used to analyze and optimize the learning process (cf. learning analytics). A review by Viberg et al. (2018) for instance shows that there is much potential for using and analyzing this data to improve the learning process, but that this rarely takes place in practice.

#### Evaluating and modifying

As well as assessing students’ learning processes, teachers in HE also need to be able to evaluate their (re)designed learning arrangements using ICT and modify their teaching practice accordingly (sub-dimension 3). To do so, teachers can use data from digital systems and digital learning resources (van Loon et al., 2018). At the same time, they should also be able to reflect on their own educational practice and design and implement improvements on how to integrate digital technologies into teaching and learning processes, and in particular the suitability of ICT for improving student learning. Based on the above, the following HE teachers’ competencies for designing, implementing, and evaluating education were formulated.

#### Validation of the dimension teachers’ digital practice

The experts stressed the importance of using the educational design cycle. Evaluating and improving digital practice should be a part of the teachers’ routines. This has been included in the third sub-dimension (see Table 1). A concern experts raised regarding design of flexible and personalized learning is that teachers do not always have control over the learning context. Experts also stressed that teacher should design their digital practice in line with their institutional educational policies. Next, they pointed to the importance of teachers’ constructive alignment in relation to the digital competencies. Teachers need to align their educational vision and beliefs on learning, subject content, learning objectives, learning activities, learning materials and educational resources, ICT use and assessment. This resulted in the first digital competence of the first sub-dimension in Table 1.

**Table 1:** Competencies for the dimension teachers’ digital practice

Designing and implementing	The teacher is able to…
	1. Design digital learning that is consistent with conceptions of teaching, the discipline and the institutional vision of education
	2. Design digital learning that responds to students’ individual needs and supports student ownership
	3. Support, combine and coordinate learning process in a variety of contexts (e.g. face-to-face, online and in the workplace)
	4. Take the well-being of students and inclusion into account in digital learning design; and
	5. Select, modify, organize and create digital resources and learning materials
Facilitating and monitoring	The teacher is able to…
	1. Use ICT to monitor and support the students’ learning process using formative and summative assessment
	2. Use ICT to collect, analyse and report on student data to understand and improve their learning process; and
	3. Use ICT to provide timely and personalized supervision and support
Evaluating and modifying	The teacher is able to…
	1. Evaluate and optimize digital learning designs
	2. Reflect on the benefits of implementing digital learning and redesign accordingly; and
	3. Reflect their digital teaching practice and adapt this to individual, institutional and societal needs

### Empowering students for a digital society

The second dimension in the HeDiCom framework focuses on empowering students for learning, working and living in a digital society (cf. Falloon, 2020). Clearly, the rapid changes taking place in society and the job market, and the related technological developments, require new digital competencies for students, also as citizens and as future employees. Fostering students’ digital literacy is the ambition of several digital competence frameworks. DigCompEdu for instance, directed to teachers at all educational levels of compulsory education, explicitly refers to “Empowering learners” as one of the six digital competence areas. This also means that teachers should empower students to use digital technologies responsibly and safely (Redecker & Punie, 2017). This resonates with the ISTE framework that states that teachers should inspire students to responsibly participate in the digital world (ISTE, 2017). Based on the comparison of the frameworks and the findings of the literature review, two sub-dimensions were identified: (1) Digital literacy for living, learning and working and (2) Digital literacy for the profession/discipline.

#### Digital literacy for living, learning and working

The digital literacy of students for living, learning and working, and the related roles of teachers in Higher Education is a key theme in the literature review (e.g., Diaconu et al., 2020; Valverde-Berrocoso & Burgos, 2017). According to Diaconu et al., (2020) HE teachers need to create and implement learning activities that enable students to develop information, media and data literacy and computational thinking. Other activities emerging from the analysis are encouraging students to create their own content or using digital technology to collaborate and communicate with others or to solve problems using digital tools (Cabero-Almenara et al., 2020; Guillén-Gámez & Mayorga-Fernández, 2020). Finally, several digital competence frameworks stressed the importance of students’ competencies to use digital technology in a safe and responsible way, to reflect about the benefits and risks of the Internet and social media, and issues about the rules and regulations governing copyright and the reuse of digital content (see e.g., JISC, 2019).

#### Digital literacy for future professions

The results of literature review clearly demonstrate that teachers in Higher Education are expected to contribute to the specific digital skills students need in their future profession (see e.g., Diaconu et al., 2020). Clearly, the digital tools that students should be familiar with vary widely, depending on the discipline. Furthermore, according to Diaconu et al. (2020), HE teachers should support students in learning how digital tools are used in their profession and teach them to critically assess their use in the future job market. Because of the rapid changes taking place in society and the further digitalization of society, the Digital Teaching Professional Framework (Education & Training Foundation, 2019) also stresses the importance of empowering students with the competencies needed to train and retrain their digital competencies throughout their careers. Based on the results, the following competencies were formulated.

#### Validation of the empowering students for a digital society dimension

In the initial draft of the framework, one of the themes was ‘fostering students’ digital literacy’. In the validation sessions, the experts suggested to replace ‘fostering students’ digital literacy’ with ‘empowering students’ digital literacy’. Empowering students is not limited to training specific digital skills, but also includes students’ digital literacy for lifelong learning, and preparing them for sustainable employment and participation in society. This was made explicit in the first sub-dimension of Empowering students for a digital society (Table 2). Furthermore, the experts advised to distinguish between digital literacy for life, learning and general work and digital literacy embedded in their future professions. They stressed the importance of developing a positive attitude towards learning about and adopting new digital technologies. A final addition to the initial framework were ethical competences related to living, learning and working in a digital society. The experts made clear that because of the GOT ethical questions became even more relevant, such as the consequences of using data. These aspects are integrated into the digital competencies in Table 2.

**Table 2:** Competencies for the dimension empowering students for a digital society

Students’ digital literacy for living, learning and working	The teacher is able to…
	1. Develop and implement learning activities for students’ digital literacy
	2. Guide students in making appropriate use of the Internet and social media
	3. Support students to effectively manage and protect personal data; and
	4. Guide students in the use of ICT to regulate and monitor their own learning process
Students’ digital literacy for future professions	The teacher is able to…
	1. Ensure that students are familiar with new digital developments in their profession/discipline
	2. Encourage students to actively contribute to new digital innovations within the profession/discipline; and
	3. Develop the digital communication skills of students to ensure continued employability

### Teachers’ digital literacy

Research evidence from the review has shown that teachers’ digital literacy is related to the quality of their educational practice using technology (see e.g., Tondeur et al., 2017) and can be considered as a prerequisite for students’ digital literacy (Falloon, 2020). Therefore, HE teachers’ and students’ digital literacy are presented in parallel in the model depicted in Fig. 1. Digital literacy is also mentioned in various frameworks, such as the Digital Teaching Professional Framework (Education & Training Foundation, 2019), the JISC Teacher profile (Higher Education) (JISC, 2019), the ISTE Standards for Educators (ISTE, 2017) and the UNESCO ICT Competence Framework for Teachers (UNESCO, 2018). Based on the comparison of these frameworks and the review of the literature, three sub-dimensions were identified: (1) digital skills, (2) Information, media and data literacy and (3) Computational thinking.

#### Essential digital skills

The first sub-dimension, essential digital skills, refers to the skills teachers in Higher Education need to use the available ICT-applications within their own educational context (Uerz et al., 2014). Some authors specifically indicate what type of applications and tools teachers should be able to use (see Ardiç & Çiftçi, 2019; Guillén-Gámez & Mayorga-Fernández, 2020; López-Belmonte et al., 2019). For example, Ardiç and Çiftçi (2019) argue that teachers should have word processing skills, spreadsheet skills, database skills, digital presentation skills, web navigation­ skills, and graphic tools skills. However, one of the criteria for this framework is to be future-proof. Consequently, the focus should shift from teachers’ skills about specific ICT applications towards the capacity to learn how to use new ICT-applications (cf. JISC, 2019; Kral et al., 2019; Tondeur et al., 2019). It can be observed that the presented competencies do not include specific tools. This was maintained in the current framework, to ensure a focus on literacies, and not on specific tools.

#### Information, media and data literacy

Based on the findings of the review, the second sub-dimension Information, media and data literacy is divided into in three foci. First, information literacy can comprise searching for information, the organization of information, and the assessment of information (Almerich et al., 2016). Carretero et al. (2017) for instance indicate that teachers must be able to analyze and compare both the information and the sources of digital content for reliability and credibility. This points to a set of competencies to perform this one task, rather than isolated competencies. Regarding media literacy, teachers, just like students, must critically reflect on the medialization of society and understand how media are created and can color one’s perception, including the risks and opportunities associated with the internet and social media (Mukhtar & Putri, 2021; van Loon et al., 2018). Finally, teachers also need to be data literate because of the increasing availability of data about students. They therefore need the necessary competencies to actively, creatively and critically use and understand data (López-Belmonte et al., 2019). These authors stated that data are part of the teaching and learning environments nowadays, i.e. they should be able to manage Big Data. This requires certain competencies in analytical treatment based on data mining, for the extraction of useful, valuable and meaningful information from large volumes of data (Huda et al., 2017).

#### Computational thinking

The third sub-dimension Computational Thinking is only recently described as a relevant competence in Higher Education (e.g., Barendsen & Bruggink, 2019; Kral et al., 2019; Tondeur et al., 2019). For teachers, this means that they need to know what computational thinking is and when it can be applied. Specifically, they need to be able to break down a complex problem into steps and processes that can be solved using digital technologies and apply these solutions in their educational practice (Barendsen & Bruggink, 2019; Lyon & Magana, 2020; Wing, 2006). Based on the above, we have defined the following HE teachers’ competencies within the digital literacy dimension.

#### Validation of the dimension teachers’ digital literacy

The literature review and the review of the existing frameworks did not result in a clear vision about the integration of teachers’ digital literacy into their general professional development. The question about whether or not to integrate these two themes was asked to the experts. Experts and practitioners stressed the importance of teachers’ digital literacy and argued to consider teachers’ digital literacy as a separate dimension as opposed to integrating it into the other dimensions. This is to make sure digital literacy receives explicit attention when it comes to HE teachers’ professional development. That is why Teachers’ digital literacy and Teachers’ professional development are two separate main dimensions (see Table 3).

**Table 3:** Competencies for the dimension teachers’ digital literacy

Essential digital skills	The teacher is able to…
	1. Effectively implement ICT in teaching practice
	2. Understand which ICT tools are appropriate in, and their impact on, educational contexts; and
	3. Actively engage with new ICT tools and technological developments
Information, media and data literacy	The teacher is able to…
	1. Locate and evaluate appropriate digital information and resources
	2. Critically engage with Internet and social media use
	3. Understand their responsibilities related to copyright, plagiarism, licensing and citation of digital resources; and
	4. Critically engage with the use of data, while protecting the personal data of students
Computational thinking	The teacher is able to…
	1. Formulate problems in their discipline using ICT
	2. Develop a solution to a problem using ICT; and
	3. Apply the solution in a specific discipline

In the draft framework, computational thinking skills were a part of teachers’ digital literacy as a separate sub-dimension. The different stakeholders agreed to this and argued that computational thinking will become more important in various types of jobs. HE teachers must be able to understand what computational thinking is in order to be able to teach this to their students. During the validation sessions, it became evident that for most participants computational thinking was a relatively new concept which needed some extra explanation and practical examples on how this could be relevant for teaching and learning. In general, for almost all of the main and sub-dimensions and competencies, participants mentioned the need for concrete and practical behavioral examples to help implement the competencies in practice.

### Teachers’ professional learning

Teachers need to continuously develop their ability to design, implement and evaluate innovative educational practices (e.g., Scherer et al., 2021; Tondeur et al., 2021). Clearly, the ability for HE teachers to keep up with technological developments in both society and in the professional discipline requires an inquisitive attitude and an ability to reflect on their own professional engagement about the role of ICT in HE. For instance, in the ISTE standards (2017), we found that teachers need to set professional learning goals, apply pedagogical approaches and then reflect on their effectiveness. Based on the comparison of digital competence frameworks and the review of the literature, three sub-dimensions were identified: (1) professional learning, (2) educational innovation with ICT, and (3) communication and collaboration.

#### Professional learning

Professional learning is an important requirement for educational innovation (Kral et al., 2019; Tondeur et al., 2019). According to Kral et al. (2019), Tondeur et al. (2019), teachers need to be able to continue to develop professionally in a manner that reflects the educational context and their own professional beliefs about education. It is also important that teachers in HE institutions work together with colleagues to develop a shared, well thought out vision and knowledge base that reflect the beliefs on teaching and learning within the team and in the university. For Almerich et al. (2016) this also means that teachers in HE should take part in research projects that focus on digital education. According to this author, teachers therefore need to apply scientific knowledge to their own teaching context and reflect on the impact of innovation on the learning processes. This brings us to the next section.

#### Innovation in digital practice

The idea that teachers need to be innovative in their use of ICT is supported by the findings of the literature review and several digital competence frameworks (Almerich et al., 2016; ISTE, 2017; Redecker & Punie, 2017). According to Scherer et al. (2021), Tondeur et al. (2021) teachers’ digital competencies in the context of educational innovation include actively following new developments in education and applying research results and best practices in the field. Moreover, HE teachers need to familiarize themselves with innovative practices by experimenting with digital technology and reflecting on their possible benefits in their teaching practice (see Martin et al., 2019).

#### Communication and collaboration

Teachers’ communication and collaboration with colleagues and other professionals are identified as important components of teachers’ digital competence in various frameworks and studies included in the review (e.g., López-Belmonte et al., 2019; Segovia Cifuentes, & Díaz Gómez, 2016). An often-mentioned example of communication is teachers’ participation in online learning networks in order to be able to learn from the wider education community (see e.g., Tondeur et al., 2018). Other examples of collaboration are design teams, learning communities or communities of practice (Alayyar et al., 2012). To illustrate, in the Tondeur et al. (2018) study teacher design teams were described as a group of two or more teachers who (re-)design—in this case—technology-enhanced curriculum materials. Based on the results, the following competencies were formulated.

#### Validation of the dimension teachers’ professional learning

Experimenting with digital technologies was already incorporated into the initial framework. But experts argued that HE teachers could do this in collaboration with other teachers, professionals and students. This was integrated into the competencies of the main dimension Teachers’ professional learning (Table 4). Furthermore, the experts pointed to the importance of teachers to be open to (innovative) digital practices and to evaluate their relevance in a systematic, research-based manner. To realize this, experts advised to provide support and time to improve their digital practice.

**Table 4:** Competencies for the dimension teachers’ professional development

Professional learning	The teacher is able to…
	1. Identify and actively develop areas of personal professional development in relation to digital innovation
	2. Work with colleagues to develop a vision for innovating and empowering students based on the institutional vision; and
	3. Evaluate their vision of innovative digital practice based on research and social trends
Innovation in digital practice	The teacher is able to…
	1. Analyse and critically evaluate digital innovation for their own practice
	2. Actively follow and critically reflect on innovative digital practice in their discipline; and
	3. Experiment with and reflect on innovative digital practice
Communication and collaboration	The teacher is able to…
	1. Collaborate with colleagues and students in the design and evaluation of innovative digital practice
	2. Participate in online communities for digital education and innovation to strengthen professional practice; and
	3. Use a range of digital technologies for communication with students, university staff and stakeholders

## Discussion and conclusion

The main goal of the current study was to create a framework of digital competencies for Higher Education that is simplified and practice focused. Based on the findings of the systematic review, the comparison of relevant existing digital competence frameworks, and the focus groups with experts and practitioners, we developed the HeDiCom Framework (see Fig. 1). This new framework includes a comprehensive set of digital competencies grouped in four dimensions: (1) Teachers’ digital practice, (2) Empowering students for a digital society, (3) Teachers’ digital digital literacy for teachers, and (4) Teachers’ professional learning. Below we discuss the main principles of the HeDiCom Framework, together with the implications for research, practice and policy. The systematic review and expert discussions confirmed these are necessary for students to develop digital competencies (see e.g., Zhao et al., 2021).

### A full picture of HE teachers’ digital competencies?

As ICT continues to drive changes in society, Higher Education institutions need to define an organizational vision in view of the planned change. In this respect, the empirical evidence stresses the importance of developing teachers’ digital competencies (see e.g., Basilotta-Gómez-Pablos et al., 2022; Bennett, 2014). As stated before, a debate exists concerning the nature of teachers’ digital competencies and how they can be best developed in Higher Education (Falloon, 2020; Tømte et al., 2015). With only four dimensions, the new HeDiCom framework is neither too complex nor too simple to provide a clear overview (see Fig. 1). Other frameworks provide a longer list of (sub-)dimensions to distinguish between types of digital competencies (e.g., DigCompEdu). According to Kimmons and Hall (2018), a good framework reduces complexity, and should be easily learned and remembered. In this respect, a too large number is less helpful to identify relevant use patterns (see also Scherer et al., 2020). This illustrates an apparent tension between the need for simplicity and the need to present a rich picture of digital competencies.

The four main dimensions in the HeDiCom framework each represent a different set of digital competencies. However, in practice differentiation between these competencies is not always straightforward. To adequately integrate the HeDiCom framework into practice requires attention to not only the separate dimensions, but also the relationship between each of them (see also Spante et al., 2018). According to Spante et al. (2018), also the assessment of a specific digital competence requires the understanding of related constructs. They are linked together in ways that make it difficult to address them separately. Further, attempting to artificially separate competencies is not necessarily useful when thinking about developing teaching and learning. To illustrate, “teachers’ digital literacy” (Dimension 4) in the HeDiCom framework can be associated with teachers' capability to “design, implement and evaluate education with ICT” (Dimension 1). Let us argue that a teacher has identified a need to develop their own level of digital literacy to better use video conferencing in their practice, to move to a more blended learning design. In this respect, Tondeur et al. (2017) stated that the distinction between digital literacy and educational technology use can be marred by the fact that technical ICT-use nevertheless involves some knowledge and skills construction. As stated before, Dimension 1—designing an ICT-rich learning environment—can also be considered as a prerequisite for empowering students for a digital society (Dimension 2). At the same time, “teachers’ professional learning with ICT” (Dimension 3) is necessary for the development of their digital literacy (Dimension 4). Therefore, these different dimensions are presented in a specific way as depicted in Fig. 1.

### The HeDiCom framework, what’s new?

Looking at the digital competencies of the HeDiCom Framework in more detail, some significant differences can be observed in comparison to existing frameworks. First of all, most digital competence frameworks were published in a pre-Covid-19 era, such as the ISTE Standards (ISTE, 2017) and the European DigCompEdu Framework (Redecker & Punie, 2017). In the new HeDiCom framework, online and blended learning came more to the front stage. Especially in the first dimension the necessary competencies to design online and blended learning environments for HE were stressed (cf. Cabero-Almenara et al., 2020; Kebritchi et al., 2017). Also fostering discipline specific and professional digital literacy for students is an aspect that was not so explicitly included in the frameworks that were analyzed in the current study. Especially in the context of Higher Education, empowering students’ discipline specific digital competencies for working can lead to professional benefits across the lifespan (Falloon, 2020).

Another relatively new digital competence addressed in the HeDiCom Framework is computational thinking, since this has received increased attention in the last few years (e.g., Barendsen & Bruggink, 2019). During the validation, experts and practitioners argued that computational thinking will become more important in jobs (Lyon & Magana, 2020). A critical issue related to computational thinking is that educational authorities in some countries, like the Netherlands, emphasize this specific innovation while others don’t. This is an example of how the dynamic and evolving relationship between technology and specific educational contexts can have clear implications on the necessary digital competencies for teachers. As a result, the aim of the HeDiCom Framework that all students must be digitally literate in order to be prepared for a knowledge-based society requires constant evaluation. Different contexts and rapid ICT-developments mean that other and new applications should also be considered within the framework. This brings us to the limitations and implications of the current study.

### Implications for practice and future research

One of the focus points of the HeDiCom Framework is facilitating the professional development for teachers in Higher Education. This new framework can serve as a common reference by providing an overview of digital competencies. In that way, the HeDiCom framework can enhance the transparency of what is expected of Higher Education teachers and support the development of their expertise (cf. McGee et al., 2017; Tondeur et al., 2019). Ultimately, improving HE teachers’ digital competencies will also improve the quality of the educational activities and the digital competencies of their students (for an overview see Zhao et al., 2021). But the assumption that ICT can facilitate and improve teaching and learning processes is often value-laden and context specific (Scherer et al., 2021; Tondeur et al., 2021). Therefore, we argue that instead of aiming for the highest levels of generalizability, we need to take into account the contextual application and the social aspects related to the adoption of digital competencies. According to Falloon (2020), the purpose of a digital competence framework is to guide curriculum design and content to the specific context in which it is to function and not precisely what it should comprise or how it should be delivered. Consequently, the use of the HeDiCom Framework should involve understanding of the historic, social, cultural, economic, and political contexts. Future research should therefore consider the implementation of the framework in relation to these societal characteristics of educational contexts.

Apart from the evaluation of the HeDiCom Framework in different contexts, more attention should be given to teachers’ digital competency development. In a best-case scenario the HeDiCom Framework can serve as a blueprint for ICT policy planning in Higher Education institutions (cf. Vanderlinde et al., 2012). To do this, the framework could be used to provide insights on the strengths and weaknesses of HE teachers’ digital competencies. This requires indicators that describe what exactly teachers should know and be able and willing to do in order to master the competencies underlying the HeDiCom sub-dimensions. Future researchers might wish to explore how these competencies can be measured. This will potentially help develop a more transparent understanding of teachers’ digital competencies and will facilitate Higher Education institutions to better train them. A concrete example of such an instrument in the context of compulsory education is SELFIE, an online tool based on the DigCompEdu framework that aims to help schools diagnose, reflect and take actions on their use of digital technologies in different areas (Castaño Muñoz et al., 2022). In order to further study teachers’ digital competence development, research should also adopt an iterative approach in developing the framework (see e.g., Ilomäki et al., 2016). As discussed above, ICT is difficult to grasp as a static concept. Rapid technological developments mean that new digital applications can also be considered within the framework. Following this line of argument, comparisons should be made over time. This makes it possible to explore influencing factors at different stages of development.

## Conclusion

The main goal of the current study was to create a framework for digital competencies in Higher Education. The resulting HeDiCom framework will enhance the transparency of what is expected of Higher Education teachers and hence support the development of their digital competencies. Ultimately, improving their digital competencies will also enhance the quality of the educational activities and the digital competencies of the students. In this study, we especially focused on the iterative construction of a comprehensive set of digital competencies and although future research is needed to further develop this framework, we hope that the HeDiCom framework can be helpful for the development of Higher Education teachers and students’ digital competencies for the future.
